# Episomal HPV16 responsible for aggressive and deadly metastatic anal squamous cell carcinoma evidenced in peripheral blood

**DOI:** 10.1038/s41598-021-84110-2

**Published:** 2021-02-25

**Authors:** Hélène Péré, Raphael Vernet, Simon Pernot, Juliette Pavie, Nicolas Robillard, Julien Puech, Sonia Lameiras, Marie-Laure Lucas, Alain Nicolas, Cécile Badoual, Bastien Rance, Laurent Bélec, Laurence Weiss, Maxime Wack, David Veyer

**Affiliations:** 1 Laboratoire de virologie, Hôpital Européen Georges Pompidou, Assistance Publique-Hôpitaux de Paris, 20-40 rue Leblanc, 75015 Paris, France; 2 INSERM U970, PARCC, Hôpital Européen Georges Pompidou, Faculté de Médecine, Centre université de Paris, Paris, France; 3 Département d’Informatique Médicale, Biostatistiques et Santé Publique, Hôpital Européen Georges Pompidou, Assistance Publique-Hôpitaux de Paris, Paris, France; 4 Oncologie médicale, Institut Bergonié, Unicancer, Bordeaux, France; 5 Service d’Immunologie clinique, Hôtel Dieu, Assistance Publique-Hôpitaux de Paris, Paris, France; 6 Institut du Fer à moulin, Inserm UMRS 839, Université Pierre et Marie Curie, Paris, France; 7 Plateforme NGS-ICGex, Institut Curie, SIRIC, Paris, France; 8 Institut Curie, Centre de Recherche, CNRS UMR3244, Université PSL, Paris, France; 9 Laboratoire d’anatomie et cytologie pathologique, Hôpital Européen Georges Pompidou, Assistance Publique-Hôpitaux de Paris, Paris, France; 10 Centre de Recherche des Cordeliers, INSERM, UMRS 1138, Université de Paris, Paris, France; 11 Inserm U976 HIPI, Université de Paris, Paris, France; 12 Unité de Génomique Fonctionnelle des Tumeurs Solides, Centre de Recherche des Cordeliers, INSERM, Université Paris, Paris, France

**Keywords:** Tumour virus infections, Tumour biomarkers, Biomarkers, Translational research

## Abstract

Archival tissue samples collected longitudinally from a patient who died from HPV16-induced high-grade anal intraepithelial squamous cell carcinoma with vertebral HPV16–positive metastasis were retrospectively analyzed by the Capture-HPV method (Capt-HPV) followed by Next-Generation Sequencing (NGS). Full length nucleotide sequences of the same HPV16 were identified from the initial and second anal biopsy samples, from plasma sample and from vertebral metastasis biopsy. Remarkably, HPV was episomal in each sample. The HPV genome sequence was closest to the HPV16 Qv18158E variant subtype (A1 lineage) exhibiting base substitutions and deletions in 7 and 2 HPV loci, respectively. In conclusion, the powerful Capt-HPV followed by NGS allows evidencing the detailed cartography of tumoral and circulating HPV DNA, giving rise to a unique and unexpected episomal virus molecular status in a context of aggressive carcinoma, underlying the importance of HPV status and its association with clinical features for further prospective studies.

## Introduction

Despite being a rare cancer accounting for approximately 20,000 new cases each year globally, the incidence of anal squamous cell carcinoma (ASCC) has been increasing dramatically over the past decade in HIV-positive patients^1^. Although the natural history of human papillomavirus (HPV) infection in ASCC remains unclear, it has been shown that high-risk HPV16 was by far the most carcinogenic genotype for ASCC^1^.

New tools and biomarkers to assess the prognosis and to improve patient stratification as well as identifying therapeutic targets are needed. Recently, sensitive double capture-HPV method (Capt-HPV) followed by Next-Generation Sequencing (NGS) allowed the capture of 208 variants from 88 HPV genotypes and thus determining at once HPV genotype and nucleotide sequence, the molecular status (episomal *versus* integrated) as well as the integration sites in both HPV and human genomes^2^.

**Figure 1:**
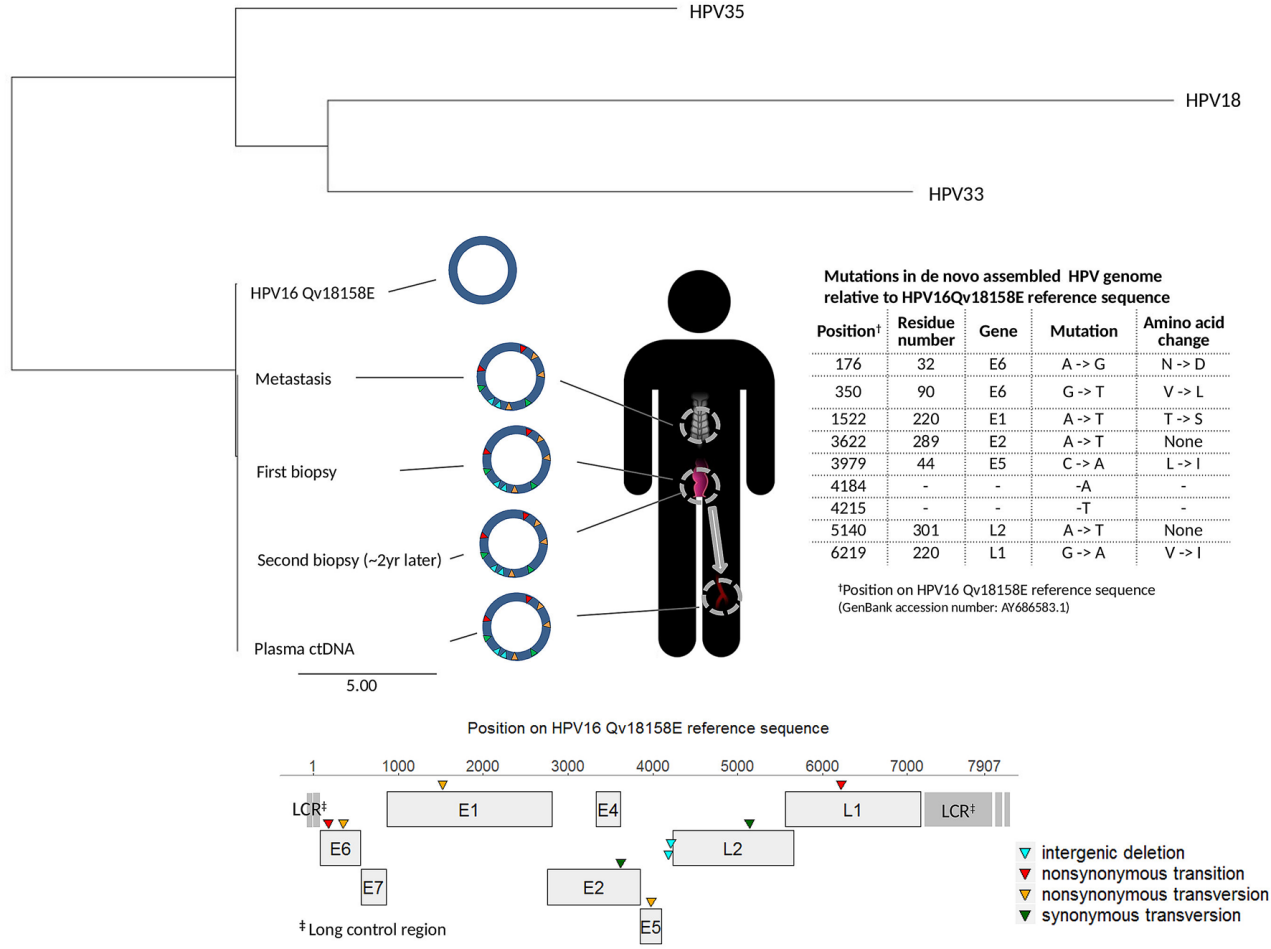
Phylogenetic tree using the neighbor joining method including the 4 patient HPV full length nucleotide sequences from the initial (June 2016) and second (July 2018) anal biopsy samples, from plasma sample (May 2018) and from vertebral metastasis biopsy (July 2018) and the HPV16 Qv18158E, HPV18, HPV33 and HPV35 reference sequences. The human junction positions are expressed relative to reference human sequence GRCh37/hg19. The scale refers to the distance between sequences. The 4 patient HPV16 full length sequences were episomal without any integration in the human genome. All 4 patient HPV sequences showed 7 nucleotide mutations by reference to HPV16 Qv18158E, including 2 nonsynonymous transitions, 3 nonsynonymous transversions and 2 synonymous transversions, and 2 nucleotide deletions, whose positions, genes and amino acid changes are depicted by reference to the HPV16 Qv18158Esequence.

We previously reported the contribution of digital droplet polymerase chain reaction for early detection of HPV-induced aggressive metastatic anal cancer in an HIV-positive MSM (Fig. 1)^3^. In brief, 18 months after the initial diagnosis and resection of an HPV16-induced high-grade anal intraepithelial neoplasm (HGAIN), the patient hospitalized at Hôpital Européen Georges Pompidou (HEGP), Paris, presented vertebral HPV16–positive metastasis, associated with a paravertebral collection. The patient received first line palliative chemotherapy with docetaxel, cisplatin, and 5FU (DCF), for 6 months, leading to stability according to RECIST criteria^4^. Due to grade 3 asthenia, a second line chemotherapy with weekly paclitaxel and carboplatin was started. After 2 months, CT-scan imagery showed progression according to RECIST criteria, and the patient presented with an ECOG performance status of 3, leading to discontinuation of chemotherapy. He received supportive care for 4 additional months and died 12 months after the first round of chemotherapy. Witnessing this very aggressive and unexpected clinical presentation prompted us to evaluate the molecular status of the HPV16 detected in the collected samples from different origins using Capt-HPV and NGS, including the initial anal biopsy that diagnosed HGAIN, a 2-year post-diagnosis follow-up anal biopsy showing HGAIN recurrence, the vertebral metastasis biopsy, and the plasmatic HPV16 ctDNA.

## Results

We identified similar HPV16 sequences with full genome coverage in the initial and second anal lesions, the plasma sample, and the metastasis biopsy. This HPV sequence was classified into the A1 lineage. The closest variant in our reference data base was HPV16 Qv18158E (GenBank accession number: AY686583.1). The virus clearly appeared episomal in each sample, without any chimeric HPV-human genome reads potentially resulting from HPV integration in the human genome. De novo assembly of the NGS reads allowed circularizing the genome, confirming its episomal status (Fig. 1). To note, our previous Capt-HPV experiments performed on ctDNA cervical tumor cases readily detected HPV integration signatures^2^, but the present study is the first one to describe episomal HPV16 ctDNA in peripheral blood.

Compared to the closest HPV16 Qv18158E genome reference, the present HPV genome analysis revealed 7 mutations. Namely, 2 base substitutions at nucleotide positions 176 and 350 were located in the *E6* coding region resulting in the simultaneous N32D and V90L amino-acid changes. Single base substitutions at positions 1522, 3979 and 6219 created E1*-*T220S, E5-L44I and L1*-*V220I mutant proteins, respectively. The remaining mutations at positions 3622 (*E2* gene) and 5140 (*L2* gene) were silent at the protein level while the nucleotide deletions at positions 4184 and 4215 were located in the same intergenic region. No mutations in the E7 and E4 genes nor in the long control region were identified.

## Discussion

Our observations report the comprehensive longitudinal molecular characterization of HPV DNA sequences from initial aggressive anal tumor to ctDNA extracted from plasma and deadly metastasis. The present study is the first one to describe episomal HPV16 ctDNA in peripheral blood. Indeed, and unexpectedly, the HPV status of each full length HPV16 sequence evidenced in tumor tissue samples as well as plasma ctDNA from study patient was episomal.

Mirabello et al*.* showed that the HPV16 E7 protein leading to cervical cancer was invariant^5^. Accordingly, we did not find any of the 32 mutations they identified in the E7 ORF from their control subjects. However, the E6-G350T found in our patient was associated with good prognosis in cervical cancer^6^, and none of the other mutations we found were previously described, underlying the need to aggregate HPV complete sequences associated with clinical features in order to correlate the presence of specific mutations with prognosis.

Capt-HPV was used previously to confirm the metastatic nature of a tongue carcinoma in a patient but no ctDNA data were provided and the same signature for HPV integration was found in both the tumor and the metastasis^7^. Using Capt-HPV, we also recently showed that 2 out of 4 patients with metastasis after an initial ASCC presented episomal HPV in their initial tumor, but no data were available regarding ctDNA nor metastasis, and de novo assembly was not performed to confirm the episomal status neither^8^. Our observation that episomal HPV can lead to metastatic progression challenges the widespread claim that tumors with integrated HPV are of worse prognosis than the ones with episomal HPV. Furthermore, HPV variants remained unchanged over time in this very aggressive metastatic ASCC, raising the central issue to which extent the cancer-associated HPV virus was functionally active during the advance of the disease and the therapeutic treatments or remained passive once the oncogenic process had started.

The present case report highlights that the availability of robust, affordable and reliable technologies such as Capt-HPV followed by NGS should facilitate the design of prospective studies where HPV status and its complete DNA sequence at baseline and/or during the follow-up would be associated with clinical data, especially to anticipate overall and progression-free survival and to offer the best adapted personalized treatment.

## Methods

DNA extracts were obtained from formalin-fixed and paraffin-embedded anal and vertebral biopsies as well as from HPV16 circulating tumoural positive plasma, as described^9^. Capt-HPV was performed using the SeqCap EZ library reagents (Roche NimbleGen)^2^. Illumina MiSeq systems were used to sequence the post capture libraries (150 bp paired-end reads). NGS data were automatically analyzed using a specific pipeline developed by the HEGP biomedical informatics team. HPV genotyping, HPV full length sequence, HPV molecular status, and positions of HPV-human junctions when present were obtained. To confirm the assumption that HPV was episomal when no integrations sites were found, we performed de novo assembly of the virus using the software Geneious (Auckland, New Zealand). This software was also able to circularize the HPV genome if episomal, confirming its structure. The assembled genome was aligned against our 208 reference HPV sequences in order to identify the closest variant. The closest variant lineage was assessed by building a phylogenetic tree including the assembled genomes and 10 HPV16 A, B, C and D variant lineage reference sequences^10^.

### Ethics approval

Our report is an observational study, in which the biological analyses were carried out with the patient approval before his death. Their post-mortem interpretation and publication are not in the scope of an ethical committee, according to the French Jardé’s law on biomedical research (https://www.legifrance.gouv.fr/affichTexte.do?cidTexte=JORFTEXT000032719520&categorieLien=id).

### Regulation of animal care statement

No animal was done for the study.

## Abbreviations

ASCC: Anal squamous cell carcinoma
Capt-HPV: Capture-HPV method
ctDNA: Circulating tumor DNA
DCF: Docetaxel, cisplatin, and 5FU
ECOG: Eastern Cooperative Oncology Group
GRCh: Genome reference consortium human
HEGP: Hôpital Européen Georges Pompidou
HGAIN: High-grade anal intraepithelial neoplasm
HPV: Human papillomavirus
MSM: Men who have sex with men
NGS: Next-generation sequencing
RECIST: Response evaluation criteria in solid tumors
